# Hemorrhoidal Artery Ligation (HAL) vs. Rubber Band Ligation (RBL) for Second- and Third-Degree Hemorrhoids: A Systematic Review and Meta-Analysis

**DOI:** 10.7759/cureus.79810

**Published:** 2025-02-28

**Authors:** Sabry Abounozha, Rashid Ibrahim, Tamer Saafan, Sami Mohammed, Yousif Aawsaj, Ali Yasen Mohamedahmed

**Affiliations:** 1 Colorectal Surgery, Sunderland Royal Hospital, Sunderland, GBR; 2 General Surgery, Wythenshawe Hospital, Manchester, GBR; 3 General Surgery, Cumberland Infirmary, Carlisle, GBR; 4 Urology, Glangwili General Hospital, Carmarthen, GBR; 5 Colorectal Surgery, Northumbria Healthcare NHS Foundation Trust, Newcastle upon Tyne, GBR; 6 General Surgery, University Hospitals Coventry and Warwickshire, Coventry, GBR

**Keywords:** 2nd and 3rd degree hemorrhoids, 2nd degree and 3rd degree haemorrhoids, hemorrhoidal artery ligation operation (halo), hemorrhoids surgery, proctology, rubber band ligation procedure

## Abstract

This systematic review investigates the outcomes of rubber band ligation (RBL) vs. hemorrhoidal artery ligation (HAL) for second and third-degree hemorrhoids. This review was designed, performed, and reported as per the recommendations of the Cochrane Handbook for Systematic Reviews of Interventions and Preferred Reporting Items for Systematic Reviews and Meta-Analyses (PRISMA) guidelines. Literature databases, including PubMed, Cochrane, Science Direct, and Google Scholar, were searched for studies comparing rubber band ligation vs. hemorrhoidal artery ligation for second- and third-degree hemorrhoids. The primary outcome was the recurrence of hemorrhoids, while post-operative bleeding, post-operative pain, surgical site infection, and success rate were the secondary outcomes. The literature search and inclusion criteria identified five studies (n=953) comparing HAL (n=548) vs. RBL (n=405). The recurrence rate was higher in the RBL group (28.4%) compared to the HAL group (19.3%) (odds ratio {OR}: 0.57, p=0.001). The two groups showed comparable results regarding post-operative pain (OR: 0.77, p=0.77), post-operative bleeding (OR: 1.48, p=0.44), and surgical site infection (risk difference: 0.00, p=0.67). Moreover, the short-term success rate was 85% in HAL compared to 86% in RBL (p=0.71). Rubber band ligation and hemorrhoidal artery ligation showed comparable short-term outcomes regarding symptom treatment, post-operative bleeding, and pain. However, HAL was superior in terms of recurrence rate.

## Introduction and background

Hemorrhoids arise from the enlargement of the hemorrhoidal plexus and pathological changes in the anal cushions of the anal canal. It is a common disease affecting 13-36% of the general population [1,2], and it is estimated that over 20000 hemorrhoidal surgeries are done in the United Kingdom yearly [3]. Internal hemorrhoids are divided into grades 1-4 according to the degree of protrusion and prolapse [4,5]. Symptoms of hemorrhoids include bleeding, pain, itching, swelling, and prolapse, and patients with hemorrhoids may need repeated hospital visits and treatment, which causes community and medical system burden [6]. Treatment of the hemorrhoids depends on the severity of symptoms and prolapse, which range from dietary and lifestyle changes, outpatient procedures such as rubber band ligation, infrared coagulation, or sclerotherapy to more invasive hemorrhoidal artery ligation (HAL) procedure, hemorrhoidectomy, or stapled hemorrhoidectomy [7-9]. Rubber band ligation (RBL) and HAL are commonly performed in the United Kingdom for grades 2 and 3 hemorrhoids. RBL is an easy, cheap, outpatient procedure that can be repeated multiple times; however, 30% of the patients develop recurrent symptoms after basic treatment and repeat RBL [10]. HAL is a more invasive alternative treatment that requires anesthesia and is performed under Doppler guidance to identify and suture ligate the hemorrhoidal artery. Sometimes, mastopexy is done in the same setting.

There has been a debate about the best procedure for the treatment of grades 2 and 3 hemorrhoids. European Society of Coloproctology recommends that RBL should be used in grades 1-3 hemorrhoids and repeat banding may be necessary (moderate level of evidence). Doppler-guided hemorrhoid artery ligation ± mucopexy could be used in patients with grades 2 and 3 hemorrhoids and/or in patients who are refractory to outpatient procedures (low level of evidence) [11].

Multiple studies have compared the efficacy of RBL vs. HAL for grades 2 and 3 hemorrhoids. This study is a systematic review and meta-analysis aiming to compare the outcomes of RBL and HAL for grades 2 and 3 hemorrhoids.

## Review

Methods

This review was designed and carried out following the recommendations of the Cochrane Handbook for Systematic Reviews of Interventions and the Preferred Reporting Items for Systematic Reviews and Meta-Analyses (PRISMA) guidelines [12,13]. The studies included in this review followed the Population, Intervention, Comparator, Outcomes (PICO) framework, which we also applied in our study as described further. The population consisted of patients undergoing hemorrhoid treatment. The intervention was hemorrhoidal artery ligation (HAL), while the comparator was rubber band ligation (RBL). The primary outcome was post-operative recurrence, defined as the recurrence of symptoms within 12 months. Secondary outcomes included post-operative bleeding, defined as bleeding requiring at least admission and observation; post-operative pain, assessed using a 10 cm visual analog scale; surgical site infection, requiring at least antibiotic therapy; and success rates, defined as the resolution or improvement of symptoms for at least six months.

Study Design

This systematic review and meta-analysis included comparative studies only. Case series, case reports, letters to the editor, and non-comparative single-arm studies were excluded. Data from conference abstracts available online were included as well.

Search Strategy

A thorough search was performed across several online databases and clinical trial registries, including PubMed, MEDLINE, ScienceDirect, Embase, Scopus, ClinicalTrials.gov, and the Cochrane Central Register of Controlled Trials (CENTRAL). The search included studies up to October 10, 2024, and was restricted to human studies published in English. Additionally, a manual search of reference lists and bibliographies from previous reviews was conducted to find supplementary studies.

We used a combination of the following MeSH terms for the search: “Hemorrhoidal artery ligation” OR “HAL” OR “THD” AND “Rubber band ligation” OR “RBL” AND “Treatment Outcome.” Two independent reviewers conducted the search and screened the extracted articles.

Eligibility and Study Selection Criteria

The articles included in this analysis were selected based on the PICO(s) framework described above. Studies that evaluated other techniques were excluded. Duplicate studies were removed, and two reviewers independently screened the titles and abstracts of the selected articles to assess their relevance. Articles were categorized as included, excluded, or requiring further evaluation. Full texts of studies that met the inclusion criteria were retrieved for further review. Any disagreements regarding study selection were resolved through discussion. If disagreements persisted, the authorship team was consulted to reach a consensus.

Data Extraction and Collection

An electronic spreadsheet, designed in accordance with Cochrane recommendations, was used for data extraction. After pilot testing with a random selection of articles, the spreadsheet was refined to create the final version. Two reviewers independently extracted key information from each study, including study-related details such as authors, publication year, location, study design, number of patients in each group, and inclusion/exclusion criteria, as well as baseline demographic and clinical characteristics of the study population. They also extracted data on primary and secondary outcomes. Any disagreements during data extraction were resolved through discussion between the reviewers, and unresolved issues were referred to the authorship team for final resolution.

Assessment for Risk of Bias

The Cochrane risk of bias tool was used to appraise the risk of bias for the randomized trials [14]. Two investigators independently reviewed all studies and graded the risk as "high," "low," or "unclear" in the following categories: random sequence generation, allocation concealment, blinding of participants and personnel, blinding of outcome assessment, incomplete outcome data, selective reporting, and other sources of bias. Regarding observational studies, the methodological quality and risk of bias assessment were carried out by two authors using the Newcastle-Ottawa scale (NOS) [15]. The NOS is a star-based scoring system (maximum score 9) that enables review authors to evaluate an observational study in the following aspects: the selection of the study groups, the comparability of the groups, and the ascertainment of the outcome of interest. Studies with a score of 9 stars were considered at low risk of bias, studies with a rating of 7 or 8 stars were considered at medium risk, and those that scored 6 or less were judged to be at high risk of bias. Discrepancies were resolved by discussion, and if they remained unresolved, a third investigator was consulted.

Data Synthesis and Statistical Analyses

The odds ratio (OR) was estimated for dichotomous outcomes. The OR is the risk of an adverse event in the HAL group compared to the RBL group. An OR of less than one would favor the HAL. Mean differences (MD) with 95% CI were used for continuous outcomes. When mean values were not available for continuous outcomes, data on median and interquartile range (IQR) were extracted and subsequently converted to mean and standard deviation (SD) using the well-practiced equation described by Hozo et al. [16].

The results were considered statistically significant at p<0.05 level or if the 95% CI did not include 1. The Cochran Q test (χ^2^) was used to evaluate heterogeneity, and I^2^ was reported to quantify it; a value of 0% indicated no heterogeneity, and over 75% indicated significant heterogeneity. A fixed effect model was used unless heterogeneity is ≥75%, whereas a random effect model was applied. Results were considered statistically significant if p<0.05. We did not generate funnel plots to assess publication bias for the reported outcomes, as the number of included studies was only five, which is below the minimum requirement for this analysis [17]. All statistical analyses were done using RevMan 5.3 (London, United Kingdom: Cochrane Collaboration).

We conducted sensitivity analyses to explore potential sources of heterogeneity and assess the robustness of our results. We repeated the primary analysis for each outcome parameter using random or fixed-effect models. Moreover, we calculated the pooled odds ratio (RR) or risk difference (RD) for each of our defined dichotomous variables. Finally, we evaluated each study's effect on the overall effect size and heterogeneity by repeating the analysis, excluding one study at a time.

Results

**Figure 1:**
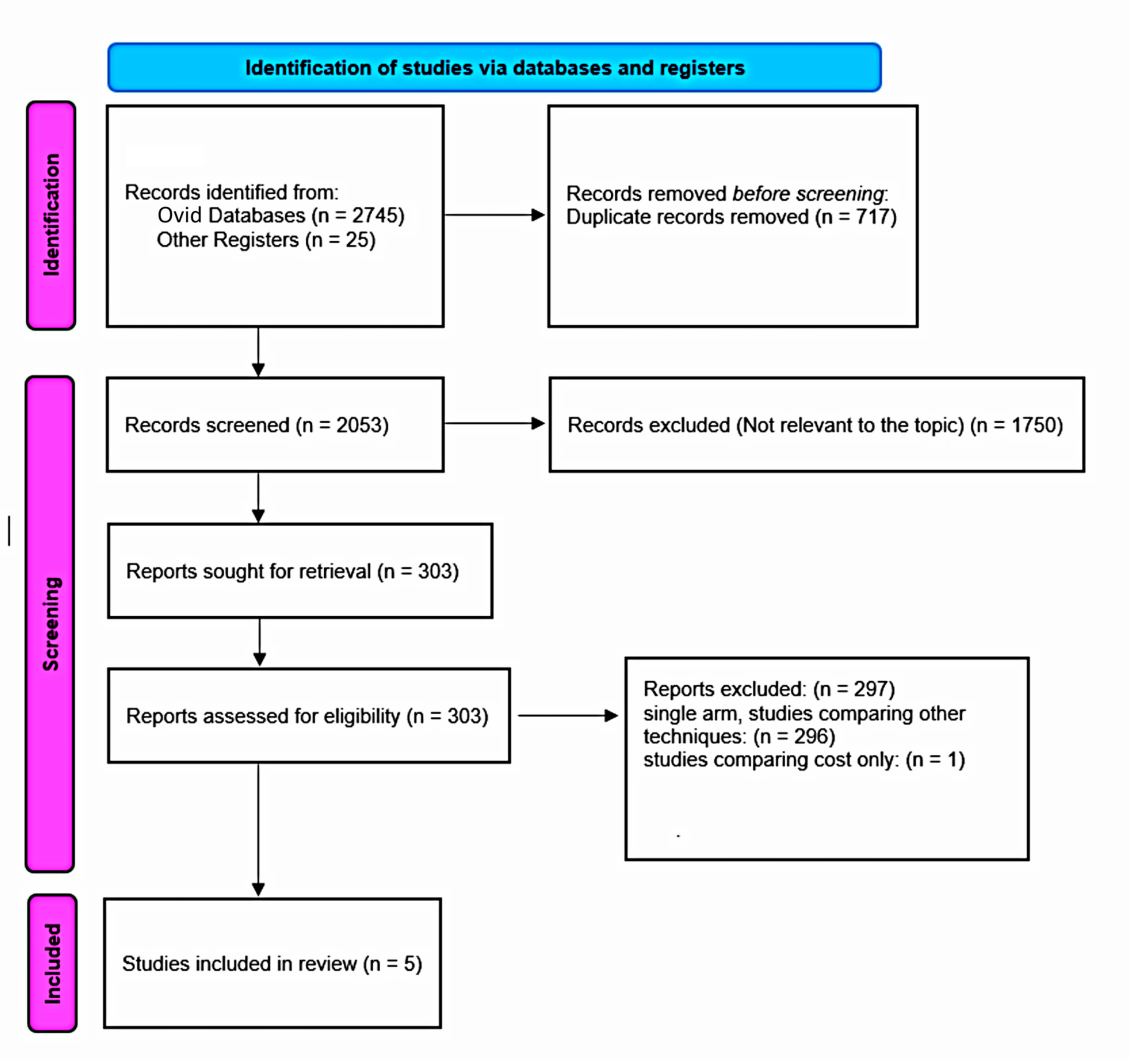
Preferred Reporting Items for Systematic Reviews and Meta-Analyses (PRISMA) flow chart.

The initial search of Ovid databases and other registers identified 2,770 records, with 2,745 from OVID and 25 from additional registers. After removing 717 duplicates, 2,053 records were screened for relevance. Of these, 1,750 were excluded as they were not pertinent to the study’s objectives. This left 303 full-text articles to be assessed for eligibility. Of these, 297 were excluded - 296 were single-arm studies comparing different techniques, and one focused solely on cost comparisons. Ultimately, five studies met the inclusion criteria and were incorporated into this review [10,18,19-21]. The PRISMA flowchart outlines this process (Figure 1).

In total, 953 patients were analyzed - 548 underwent hemorrhoidal artery ligation (HAL) and 405 underwent rubber band ligation (RBL). Of the five studies included, two were randomized controlled trials (with a total of 472 patients), and the remaining three were observational studies. Detailed information about study design, sample sizes, hemorrhoid grades, inclusion and exclusion criteria, follow-up periods, and procedural techniques are provided in Tables 1, 2.

**Table 1:** Characteristics of included studies. HAL: hemorrhoidal artery ligation; RBL: rubber band ligation; NA: unavailable; RCT: randomized controlled trial; IBD: inflammatory bowel disease; AMI: Agency for Medical Innovations THD Slide One (Correggio, Italy: THD S.p.A.)

Studies	Country	Type of the study	Number of patients	Inclusion and exclusion criteria	Anesthesia	HAL device
Agrawal et al. (2022) [18]	India	RCT	HAL: 50, RBL: 50	Inclusion criteria: grades 2 and 3 hemorrhoids. Exclusion criteria: grades 1 and 4 hemorrhoids, comorbid or previous anorectal surgery.	NA	NA
Shehata et al. (2019) [19]	Egypt	Prospective observational study	HAL: 24, RBL: 25	Inclusion criteria: grades 2 and 3 hemorrhoids. Exclusion criteria: grades 1 and 4, concomitant anal disease, IBD, hematological disorders, anticoagulation, previous anorectal surgery.	HAL: spinal RBL: none	THD Slide One
Yılmaz et al. (2017) [20]	Turkey	Retrospective	HAL: 50, RBL: 96	Inclusion criteria: grades 2 and 3 hemorrhoids. Exclusion criteria: grades 1 and 4 hemorrhoids.	HAL: local RBL: topical	THD Slide One
Brown et al. (2016) [10]	United Kingdom	RCT	HAL: 185, RBL: 187	Inclusion criteria: grades 2 and 3 hemorrhoids. Exclusion criteria: previous hemorrhoid surgery, perianal sepsis, inflammatory bowel disease, colorectal malignancy, pre-existing sphincter injury, immunodeficiency, and hypercoagulability disorders.	NA	THD Slide One and AMI
Pol et al. (2011) [21]	Netherlands	Prospective observational study	HAL: 239, RBL: 47	Inclusion criteria: grades 1 (if failed conservative treatment) 2 and 3 hemorrhoids. Exclusion criteria: grades 4 and grade 1 (if successful conservative treatment) hemorrhoids.	HAL: epidural RBL: epidural	AMI

**Table 2:** Baseline characteristics of the included population. HAL: hemorrhoidal artery ligation; RBL: rubber band ligation; NA: unavailable; All: both HAL and RBL groups

Studies	Age in years (mean±SD)	Gender (male: female)	Follow-up period (months)	Garde 2 hemorrhoids	Grade 3 hemorrhoids
Agrawal et al. (2022) [18]	All: 52.4±11.7	NA	All: 12	All: 80	All: 20
Shehata et al. (2019) [19]	HAL: 45.4±14.2, RBL: 42.3±13.3	HAL: 18:7, RBL: 15:10	All: 6	HAL: 15, RBL: 11	HAL: 10, RBL: 14
Yılmaz et al. (2017) [20]	HAL: 26.4, RBL: 24.8	HAL: 21:29, RBL: 72:24	All: 6	HAL: 22, RBL: 41	HAL: 28, RBL: 55
Brown et al. (2016) [10]	HAL: 48.5±13.5, RBL: 49±12.9	HAL: 85:76, RBL: 99:77	All: 12	HAL: 92, RBL: 115	HAL: 68, RBL: 60
Pol et al. (2011) [21]	HAL: 49, RBL: 57	HAL: 149:90, RBL: 24:23	HAL: 24.8, RBL: 19.9	HAL: 116, RBL: 19	HAL: 95, RBL: 21

Risk of bias assessment

**Figure 2:**
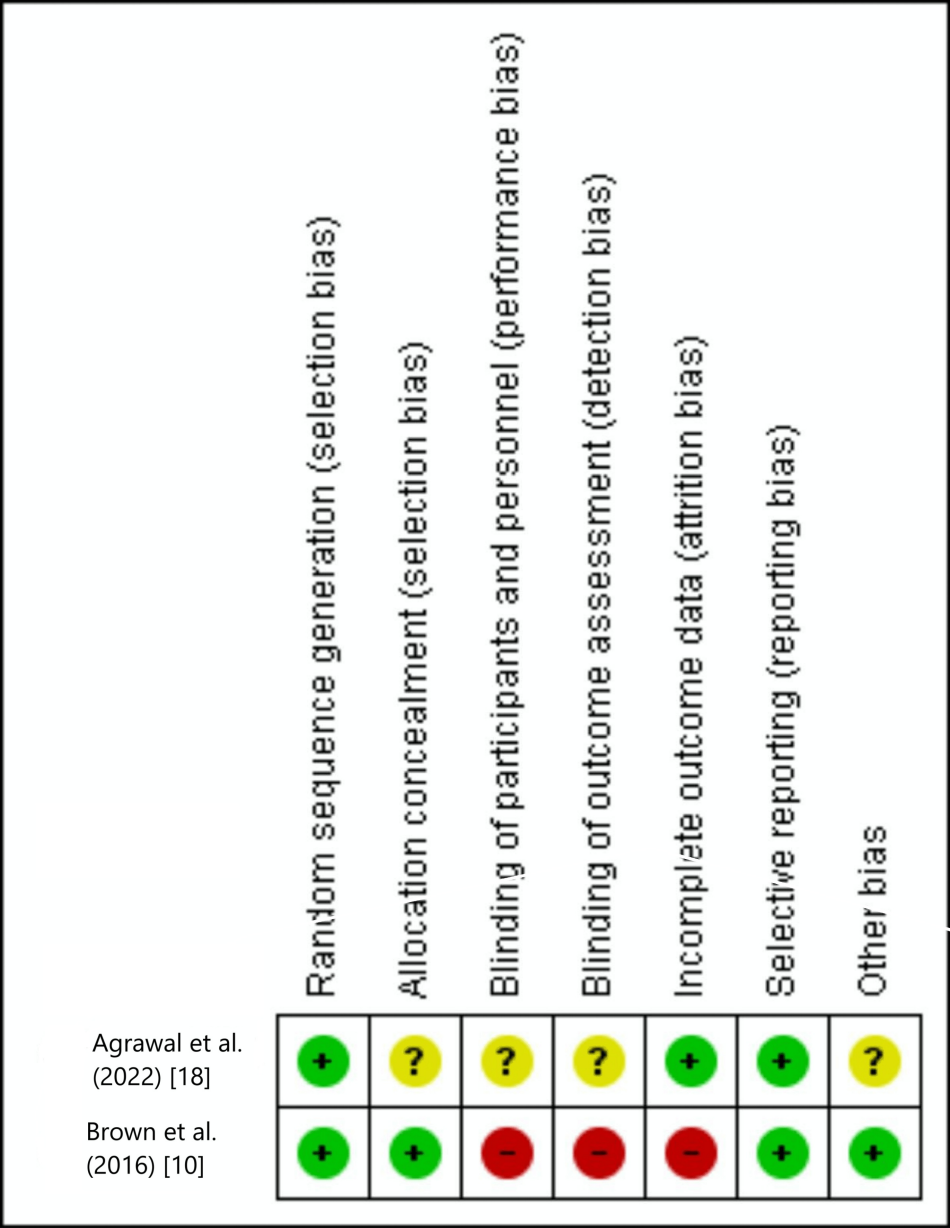
Risk of bias summary of included RCTs.

**Figure 3:**
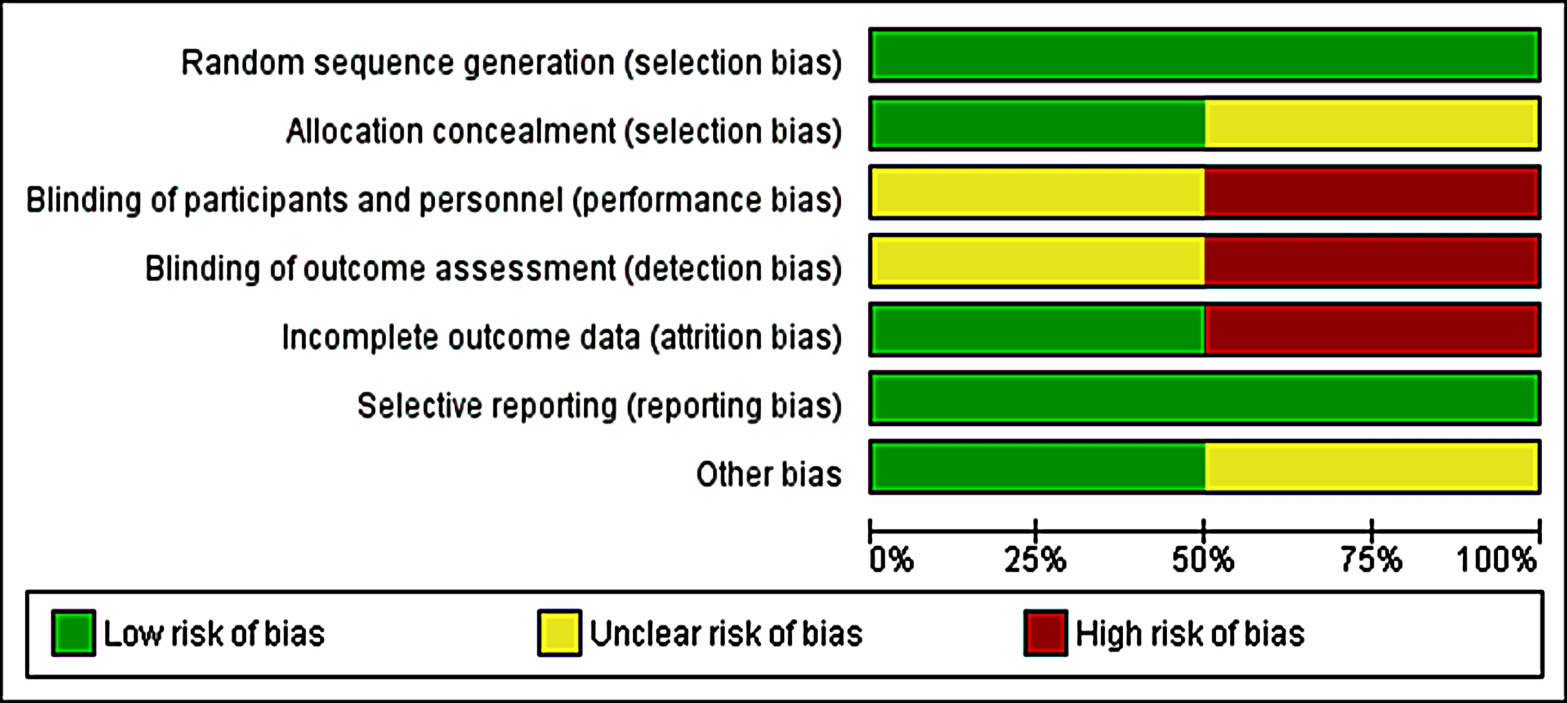
Risk of bias graph of included RCTs.

Table 3 highlights the outcomes of methodological quality assessment based on the NOS for observational studies. The risk of bias assessment for the included RCTs is shown in Figures 2, 3.

**Table 3:** Risk of Bias assessment of the observational studies with the Newcastle-Ottawa system. The NOS is a star-based scoring system (maximum score 9) that enables review authors to evaluate an observational study. The overall score is used to determine the risk of bias. A score of 7-9 stars indicates a low risk of bias, 4-6 stars indicates an unclear risk of bias, and 3 or fewer stars indicates a high risk of bias.

Studies	Representativeness of the exposed cohort	Selection of the non-exposed cohort	Ascertainment of exposure	Demonstration that outcome of interest was not present at start of the study	Comparability of cohorts based on the design or analysis controlled for confounders	Assessment of outcome	Was follow-up long enough for outcomes to occur	Adequacy of follow-up of cohorts	Total
Pol et al. (2011) [21]	*	*	*	*		*	*	*	7
Yılmaz et al. (2017) [20]	*	*	*	*		*	*	*	7
Shehata et al. (2019) [19]	*	*	*	*	*	*	*	*	8

Primary outcome

Recurrence

**Figure 4:**
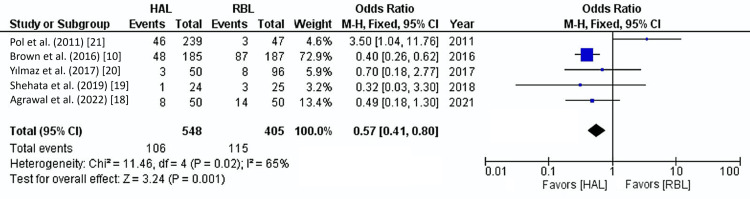
Recurrence Forest plot. HAL: hemorrhoidal artery ligation; RBL: rubber band ligation; M-H: Mantel-Haenszel fixed-effect model

All five studies reported recurrence rates across a total of 953 patients. The overall recurrence rate for both treatment groups was 23.1%. Pooled analysis revealed a significantly higher recurrence rate in the RBL group compared to the HAL group (28.4% vs. 19.3%) (OR: 0.57, 95% CI: 0.41-0.80, p=0.001). Moderate heterogeneity was observed among the studies (I²=65%, p=0.02) (Figure 4).

Secondary outcomes

Post-operative Bleeding

**Figure 5:**
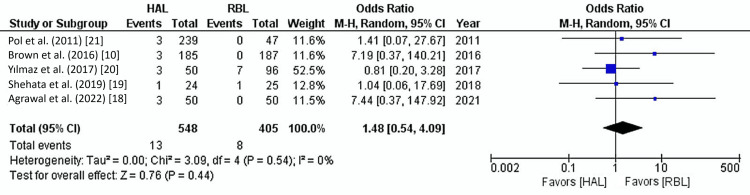
Post-operative bleeding Forest plot. HAL: hemorrhoidal artery ligation; RBL: rubber band ligation; M-H: Mantel-Haenszel fixed-effect model

All five studies reported post-operative bleeding rates for a total of 953 patients. The overall post-operative bleeding rate was 2.2%. Pooled analysis indicated no significant difference in bleeding rates between HAL and RBL groups (2.4% vs. 2%) (OR: 1.48, 95% CI: 0.54-4.09, p=0.44). The heterogeneity between studies was low (I²=0%, p=0.54) (Figure 5).

Post-operative Pain

**Figure 6:**
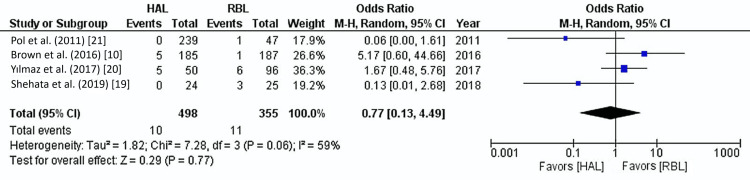
Post-operative pain Forest plot. HAL: hemorrhoidal artery ligation; RBL: rubber band ligation; M-H: Mantel-Haenszel fixed-effect model

Four of the five studies reported post-operative pain outcomes, including 853 patients. The overall post-operative pain rate was 2.7%. No significant difference was found between HAL and RBL groups regarding pain (2% vs. 3%) (OR: 0.77, 95% CI: 0.13-4.49, p=0.77). Moderate heterogeneity was observed among the included studies (I²=59%, p=0.06) (Figure 6, Table 4).

**Table 4:** Post-operative pain characteristics among different studies. HAL: hemorrhoidal artery ligation; RBL: rubber band ligation; NA: unavailable; VAS: visual analog scale

Studies	Pain			p-Value
	Assessment type	HAL	RBL	
Pol et al. (2011) [21]	NA	NA	NA	NA
Brown et al. (2016) [10]	VAS (mean score)	1st day: 4.6, 7thday: 3.1, 21st day: 1.3, and 6 weeks: 1.0	1st day: 3.4, 7thday: 1.6, 21st day: 1.4, and 6 weeks: 1.2	1st day: 0.0002, 7thday: <0.0001, 21st day: 0.44, and 6 weeks: 0.32
Agrawal et al. (2022) [18]	NA	NA	NA	NA
Shehata et al. (2019) [19]	Severe pain (number of cases)	Grade 2: 0, grade 3: 0	Grade 2: 1, grade 3: 2	Grade 2: 0.23, grade 3: 0.21
Yılmaz et al. (2017) [20]	Severe pain (number of cases)	5	6	NA

Surgical Site Infection

**Figure 7:**
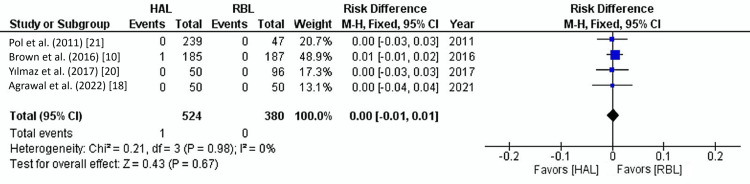
Surgical site infection Forest plot. HAL: hemorrhoidal artery ligation; RBL: rubber band ligation; M-H: Mantel-Haenszel fixed-effect model

Surgical site infection rates were reported in four studies involving 904 patients. The overall infection rate was 0.1%. There was no significant difference in infection rates between HAL and RBL groups (0.2% vs. 0%) (OR: 0.00, 95% CI: 0.01-0.01, p=0.67). The level of heterogeneity was low (I²=0%, p=0.98) (Figure 7).

Success Rate

**Figure 8:**
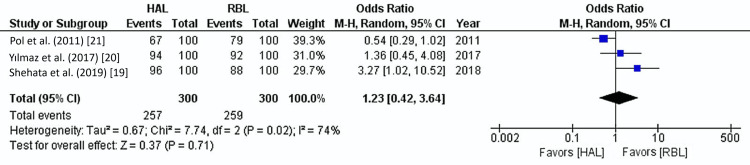
Success rate Forest plot. HAL: hemorrhoidal artery ligation; RBL: rubber band ligation; M-H: Mantel-Haenszel fixed-effect model

Three of the five studies, comprising 600 patients, reported the success rates of the procedures. The overall success rate across both treatment groups was 86%. Pooled analysis showed no significant difference in success rates between HAL and RBL (85% vs. 86%) (OR: 1.23, 95% CI: 0.42-3.64, p=0.71). A moderate level of heterogeneity was found among the included studies (I²=74%, p=0.02) (Figure 8).

Discussion

Hemorrhoid disease can significantly impact a patient’s quality of life, particularly when symptoms such as pain, bleeding, and prolapse become persistent and severe [22]. These symptoms have been linked to heightened discomfort and daily life disruptions, and hemorrhoid-specific quality of life (QoL) assessments demonstrated notable improvements in patient well-being following invasive treatments such as rubber band ligation and sclerotherapy [23]. However, other studies suggest that while symptoms can be distressing, hemorrhoids do not consistently reduce physical and mental health scores when assessed with general QoL measures. A large-scale study in Austria found no statistically significant difference in health-related quality of life scores between symptomatic and asymptomatic hemorrhoid patients using the Short Form-12 Health Survey. This suggests that treatment decisions should be carefully considered and focused on symptom relief rather than QoL outcomes alone [24]. These findings highlight that while hemorrhoids impact quality of life, targeted treatments effectively relieve symptoms and enhance patient-reported outcomes, especially when daily life is significantly affected.

There appears to be a lack of systematic reviews and meta-analyses evaluating the comparative effectiveness and safety of hemorrhoidal artery ligation (HAL) and rubber band ligation (RBL) for grades 2 and 3 hemorrhoids. This review of five studies, including a total of 953 patients, revealed notable differences in recurrence rates between HAL and RBL, with HAL showing a reduced recurrence rate compared to RBL. It is important to highlight that our study specifically focuses on recurrence after a single attempt at comparing both techniques. This finding suggests that HAL may offer a more sustainable solution for alleviating hemorrhoidal symptoms over an extended period. However, secondary metrics such as post-operative bleeding, post-operative pain, surgical site infections, and procedural success rates did not show significant differences between the two intervention techniques.

This study indicated a significantly lower recurrence rate for HAL compared to RBL, with pooled recurrence rates of 19.3% for HAL and 28.4% for RBL (odds ratio: 0.57, 95% confidence interval: 0.41-0.80, p=0.001). This suggests that HAL may provide more enduring relief from hemorrhoidal symptoms, particularly in individuals diagnosed with grades 2 and 3 hemorrhoids. By specifically targeting the hemorrhoidal arterial supply, HAL likely reduces blood flow to the hemorrhoidal tissue, in turn decreasing the likelihood of recurrence [25,26]. In contrast, RBL primarily works through mechanical ligation, which addresses immediate symptomatic relief rather than the underlying vascular supply, resulting in potentially higher recurrence rates over time [27,28]. Our observations regarding recurrence rates align with prior literature comparing HAL and RBL. Brown et al., in a large multicentre randomized controlled trial, found that the recurrence rate was significantly higher in the RBL cohort compared to the HAL group, with rates of 49% vs. 30% at one-year post-operative follow-up [10]. These results were further supported by Agrawal et al. who reported a more significant percentage of individuals requiring intervention in the RBL group than in the HAL group [18]. Interestingly, Pol et al. reported a higher recurrence rate in the HAL group compared to the RBL group, with rates of 19% vs. 6% (p<0.05) [21]. This suggests that while HAL reduces arterial inflow to the hemorrhoidal tissues, which may alleviate bleeding symptoms, it does not facilitate tissue retraction, thereby leaving mucosal prolapse unaddressed, which is the main factor in predicting disease recurrence and the need for subsequent procedural interventions [21].

Interestingly, despite the observed differences in recurrence rates, this analysis revealed no statistically significant difference between HAL and RBL regarding post-operative bleeding, pain, and surgical site infections. Both techniques exhibited comparable safety profiles, with low rates of post-operative bleeding (2.4% for HAL and 2% for RBL) and minimal post-operative pain (2% for HAL and 3% for RBL). These findings imply that both procedures are relatively safe and well-tolerated by patients. However, we must acknowledge the moderate heterogeneity evident in the post-operative pain data (I²=59%), which may indicate variability in patient pain tolerance, procedural techniques, or discrepancies in pain measurement and reporting across different studies.

In this meta-analysis, we found no significant difference in post-operative pain rates between HAL and RBL, with an overall pain rate of 2.7% across both groups (2% for HAL and 3% for RBL, odds ratio: 0.77, 95% confidence interval: 0.13-4.49, p=0.77). These results align with previous studies, which generally report low pain levels for both procedures, although each has distinct pain profiles due to differences in procedural mechanics and tissue impact [25,26,28]. Post-operative pain in RBL typically arises from localized ischemia, as the rubber band restricts blood flow, causing tissue necrosis and eventually slough off [29]. This ischemic process can result in moderate pain immediately after the procedure, often peaking within the first 24-48 hours. However, pain levels in RBL are generally mild-to-moderate and tend to subside as the tissue adjusts to the banding [28]. In contrast, pain in HAL mainly arises from the manipulation of the hemorrhoidal arteries and the placement of sutures within the anal canal. Unlike RBL, which directly targets the tissue, HAL reduces blood flow through arterial ligation, leading to less ischemic trauma and often a more tolerable post-operative experience. However, due to the suturing involved in HAL, some patients may experience low-level discomfort or mild pain, which is typically managed with standard analgesics [25,30]. Because HAL is a more targeted approach, the pain is usually transient and less severe compared to more invasive surgical treatments. Our findings align with other studies comparing post-operative pain between RBL and HAL. Brown et al. found that while patients undergoing HAL reported occasional low-level pain following arterial ligation, this was not statistically significant compared to RBL [10]. Other studies also demonstrated that the post-operative pain after the HAL procedure significantly improves after the first week of surgery [31,32].

In this study, the overall post-operative bleeding rate was relatively low for both RBL and HAL, with no statistically significant difference between the two methods (2.4% for HAL and 2% for RBL; OR: 1.48, 95% CI: 0.54-4.09, p=0.44). These findings are consistent with other studies that generally report minimal bleeding associated with both procedures [19,33]. However, some differences may arise due to the specific mechanisms of each treatment and patient factors such as hemorrhoid grade and bleeding tendency. For RBL, post-operative bleeding typically occurs due to the sloughing of necrotic tissue following the application of the rubber band to the hemorrhoid [33]. This necrosis and subsequent sloughing can lead to a small amount of bleeding as the necrotic tissue detaches from the hemorrhoidal site. Generally, this bleeding is mild and self-limiting, often resolving without intervention. However, in rare cases, bleeding can be more pronounced, particularly if the patient is on anticoagulants or antiplatelet treatment [34]. In contrast, post-operative bleeding after HAL is usually less common because the procedure focuses on ligating the hemorrhoidal arteries, thereby reducing the blood supply to the hemorrhoidal tissue instead of causing direct tissue necrosis [25,35]. Unlike RBL, HAL is not associated with sloughing tissue, so bleeding after HAL is generally minimal. When bleeding does occur, it is typically related to suture dislodgement or improper ligation; such bleeding is usually less frequent and can often be managed conservatively without further intervention [36].

In this analysis, post-operative infection rates were low across both RBL and HAL groups, with no significant difference between the two procedures, 0.2% for HAL and 0% for RBL (OR: 0.00, 95% CI: 0.01-0.01, p=0.67). The observed low heterogeneity (I²=0%) indicates consistency in infection rates across the studies. The low infection rates associated with these procedures make them attractive alternatives to more invasive surgical interventions, such as hemorrhoidectomy, which generally carries a higher risk of complications, including infections [37]. The risk of infection in RBL is low because the procedure is minimally invasive. However, rare infections can occur if the necrotic tissue caused by banding becomes secondarily infected. This can happen if the band is placed too close to the dentate line, leading to mucosal trauma or prolonged tissue necrosis. Although uncommon, infections in RBL can manifest as local abscesses and, in sporadic cases, can progress to severe conditions such as perianal sepsis [38]. In HAL, the infection risk is also low due to the procedure's nature, which involves ligating the hemorrhoidal arteries without directly exposing the tissue to the external environment. However, since HAL includes suturing within the anal canal, there is a small risk of localized infections at the suture sites, especially in patients with pre-existing risk factors like immunosuppression.

This meta-analysis highlights that procedural success rates were nearly equivalent between HAL and RBL, with an 85% success rate for HAL and 86% for RBL. This similarity suggests that both methods effectively treat grades 2 and 3 hemorrhoids, leading to a high likelihood of symptomatic relief and patient satisfaction. Our analysis showed moderate heterogeneity (I²=74%), reflecting variability in success rate reporting. This likely stems from differences in success definitions, follow-up duration, and practitioner expertise.

Although our study does not include a cost analysis, it is important to acknowledge the financial cost difference between HAL and RBL. The cost-effectiveness of hemorrhoidal artery ligation (HAL) and rubber band ligation (RBL) varies primarily due to differences in procedural complexity, recurrence rates, and long-term outcomes. RBL is generally more affordable in terms of direct costs [27-29]. As a straightforward outpatient procedure that requires minimal equipment, it can often be completed quickly, making it a cost-effective option, especially for grade 2 hemorrhoids. In contrast, HAL is a more resource-intensive procedure that requires anesthesia, a Doppler probe, and specialized suturing, which increases its upfront cost [22,25]. However, for patients with grade 3 hemorrhoids, HAL may be more cost-effective in the long term due to its lower recurrence rates [30]. Fewer recurrences translate to a reduced need for additional treatments, follow-ups, or symptom management, offsetting HAL’s initial higher cost. Comparative studies suggest that RBL is economical for patients with lower-grade hemorrhoids, while HAL may be better suited for cases with a high risk of recurrence. Cost-effectiveness could be optimized by using RBL as a first-line treatment for most grade 2 cases and reserving HAL for more complex cases where long-term outcomes and lower recurrence rates are prioritized [10,39].

Limitations

This review has a few limitations. Only five studies met inclusion criteria, limiting sample size and generalizability. Two were RCTs, and three were observational, introducing potential bias. Language restrictions may have excluded relevant non-English studies. Moderate heterogeneity in recurrence and success rates underscores the need for larger, standardized studies. Future research should include large, multicenter RCTs with standardized outcomes for pain, recurrence, and success. Long-term follow-ups are needed to assess durability, along with patient-reported outcomes on quality of life and satisfaction.

## Conclusions

In conclusion, this review shows HAL has a lower recurrence rate than RBL for grades 2 and 3 hemorrhoids, suggesting longer-lasting relief. Both procedures have similar safety and success rates, making them viable based on patient factors and resources. Further research is needed on long-term outcomes and satisfaction.

## References

[1] Lohsiriwat V (2012). Hemorrhoids: from basic pathophysiology to clinical management. World J Gastroenterol.

[2] Walsh CJ, Delaney S, Rowlands A (2018). Rectal bleeding in general practice: new guidance on commissioning. Br J Gen Pract.

[3] Brown SR (2017). Haemorrhoids: an update on management. Ther Adv Chronic Dis.

[4] Sandler RS, Peery AF (2019). Rethinking what we know about hemorrhoids. Clin Gastroenterol Hepatol.

[5] Sheikh P, Régnier C, Goron F, Salmat G (2020). The prevalence, characteristics and treatment of hemorrhoidal disease: results of an international web-based survey. J Comp Eff Res.

[6] Mohamedahmed AY, Stonelake S, Mohammed SS, Zaman S, Ahmed H, Albarade M, Hajibandeh S (2020). Haemorrhoidectomy under local anaesthesia versus spinal anaesthesia: a systematic review and meta-analysis. Int J Colorectal Dis.

[7] Cocorullo G, Tutino R, Falco N (2017). The non-surgical management for hemorrhoidal disease. A systematic review. G Chir.

[8] Burch J, Epstein D, Baba-Akbari A (2008). Stapled haemorrhoidectomy (haemorrhoidopexy) for the treatment of haemorrhoids: a systematic review and economic evaluation. Health Technol Assess.

[9] Altomare DF, Giuratrabocchetta S (2013). Conservative and surgical treatment of haemorrhoids. Nat Rev Gastroenterol Hepatol.

[10] Brown S, Tiernan J, Biggs K (2016). The HubBLe Trial: haemorrhoidal artery ligation (HAL) versus rubber band ligation (RBL) for symptomatic second- and third-degree haemorrhoids: a multicentre randomised controlled trial and health-economic evaluation. Health Technol Assess.

[11] van Tol RR, Kleijnen J, Watson AJ (2020). European Society of ColoProctology: guideline for haemorrhoidal disease. Colorectal Dis.

[12] (2025). Cochrane Handbook for Systematic Reviews of Interventions. Version.

[13] Moher D, Liberati A, Tetzlaff J, Altman DG (2010). Preferred reporting items for systematic reviews and meta-analyses: the PRISMA statement. Int J Surg.

[14] Higgins JP, Altman DG, Gøtzsche PC (2011). The Cochrane Collaboration's tool for assessing risk of bias in randomised trials. BMJ.

[15] Wells GA, Shea B, O’Connell D (2025). The Newcastle-Ottawa Scale (NOS) for assessing the quality of nonrandomized studies in meta-analysis. https://web.archive.org/web/20210716121605id_/http://www3.med.unipmn.it/dispense_ebm/2009-2010/Corso%20Perfezionamento%20EBM_Faggiano/NOS_oxford.pdf.

[16] Hozo SP, Djulbegovic B, Hozo I (2005). Estimating the mean and variance from the median, range, and the size of a sample. BMC Med Res Methodol.

[17] Macaskill P, Walter SD, Irwig L (2001). A comparison of methods to detect publication bias in meta-analysis. Stat Med.

[18] Agrawal MP, Pateriya A, Samar SK (2022). Comparative assessment of efficacy of artery ligation versus rubber band ligation for management of haemorrhoids in Southern Rajasthan. Int Surg J.

[19] Shehata AM, Saleh AF, El-Heeny AA (2019). Clinical outcome after Doppler-guided hemorrhoidal artery ligation and rubber band ligation for treatment of primary symptomatic hemorrhoids. Indian J Surg.

[20] Yılmaz İ, Karakaş DÖ, Sücüllü İ, Saydam M (2017). Grade II-III hemorrhoidal disease treatment: rubber band ligation versus hemorrhoidal artery ligation. Turk J Colorectal Dis.

[21] Pol RA, van der Zwet WC, Kaijser M, Schattenkerk ME, Eddes EH (2011). Comparison of Doppler-guided haemorrhoidal artery ligation without mucopexy and rubber band ligation for haemorrhoids. Arab J Gastroenterol.

[22] Rørvik HD, Davidsen M, Gierløff MC, Brandstrup B, Olaison G (2023). Quality of life in patients with hemorrhoidal disease. Surg Open Sci.

[23] Keong SY, Tan HK, Lamawansa MD, Allen JC, Low ZL, Østbye T (2021). Improvement in quality of life among Sri Lankan patients with haemorrhoids after invasive treatment: a longitudinal observational study. BJS Open.

[24] Riss S, Weiser FA, Riss T, Schwameis K, Mittlböck M, Stift A (2011). Haemorrhoids and quality of life. Colorectal Dis.

[25] Ratto C, de Parades V (2015). Doppler-guided ligation of hemorrhoidal arteries with mucopexy: a technique for the future. J Visc Surg.

[26] Ratto C (2014). THD Doppler procedure for hemorrhoids: the surgical technique. Tech Coloproctol.

[27] Iyer VS, Shrier I, Gordon PH (2004). Long-term outcome of rubber band ligation for symptomatic primary and recurrent internal hemorrhoids. Dis Colon Rectum.

[28] Chew SS, Marshall L, Kalish L, Tham J, Grieve DA, Douglas PR, Newstead GL (2003). Short-term and long-term results of combined sclerotherapy and rubber band ligation of hemorrhoids and mucosal prolapse. Dis Colon Rectum.

[29] Albuquerque A (2016). Rubber band ligation of hemorrhoids: a guide for complications. World J Gastrointest Surg.

[30] Verre L, Gallo G, Grassi G (2022). Transanal hemorrhoidal dearterialization (THD) for hemorrhoidal disease: an Italian single-institution 5-year experience analysis and updated literature review. Front Surg.

[31] De Nardi P, Capretti G, Corsaro A, Staudacher C (2014). A prospective, randomized trial comparing the short- and long-term results of Doppler-guided transanal hemorrhoid dearterialization with mucopexy versus excision hemorrhoidectomy for grade III hemorrhoids. Dis Colon Rectum.

[32] Schuurman JP, Rinkes IH, Go PM (2012). Hemorrhoidal artery ligation procedure with or without Doppler transducer in grade II and III hemorrhoidal disease: a blinded randomized clinical trial. Ann Surg.

[33] Forlini A, Manzelli A, Quaresima S, Forlini M (2009). Long-term result after rubber band ligation for haemorrhoids. Int J Colorectal Dis.

[34] Gülen M, Emral AC, Ege B (2024). Management of hemorrhoid rubber band ligation complications: massive rectal bleeding. Namik Kemal Med J.

[35] Karkalemis K, Chalkias PL, Kasouli A, Chatzaki E, Papanikolaou S, Dedemadi G (2021). Safety and effectiveness of hemorrhoidal artery ligation using the HAL-RAR technique for hemorrhoidal disease. Langenbecks Arch Surg.

[36] Hoyuela C, Carvajal F, Juvany M (2016). HAL-RAR (Doppler guided haemorrhoid artery ligation with recto-anal repair) is a safe and effective procedure for haemorrhoids. Results of a prospective study after two-years follow-up. Int J Surg.

[37] Kunitake H, Poylin V (2016). Complications following anorectal surgery. Clin Colon Rectal Surg.

[38] Sim HL, Tan KY, Poon PL, Cheng A, Mak K (2009). Life-threatening perineal sepsis after rubber band ligation of haemorrhoids. Tech Coloproctol.

[39] Alshreef A, Wailoo AJ, Brown SR (2017). Cost-effectiveness of haemorrhoidal artery ligation versus rubber band ligation for the treatment of grade II-III haemorrhoids: analysis using evidence from the HubBLe trial. Pharmacoecon Open.

